# *Haemophilus parasuis* (*Glaesserella parasuis*) as a Potential Driver of Molecular Mimicry and Inflammation in Rheumatoid Arthritis

**DOI:** 10.3389/fmed.2021.671018

**Published:** 2021-08-17

**Authors:** Gabriele Di Sante, Elisa Gremese, Barbara Tolusso, Paola Cattani, Clara Di Mario, Simona Marchetti, Stefano Alivernini, Maria Tredicine, Luca Petricca, Ivana Palucci, Chiara Camponeschi, Virginia Aragon, Andrea Gambotto, Francesco Ria, Gianfranco Ferraccioli

**Affiliations:** ^1^Section of General Pathology, Department of Translational Medicine and Surgery, Università Cattolica del Sacro Cuore, Rome, Italy; ^2^Division of Rheumatology, Fondazione Policlinico Universitario Agostino Gemelli—IRCCS, Rome, Italy; ^3^Division of Rheumatology, Università Cattolica del Sacro Cuore, Rome, Italy; ^4^Dipartimento di Scienze di laboratorio e infettivologiche, Fondazione Policlinico Universitario A. Gemelli Istituto di Ricovero e Cura a Carattere Scientifico, Rome, Italy; ^5^Dipartimento di Scienze Biotecnologiche di Base, Cliniche Intensivologiche e Perioperatorie, Sezione di Microbiologia, Università Cattolica del S. Cuore, Rome, Italy; ^6^Institut de Recerca i Tecnologies Agroalimentaries, Centre de Recerca en Sanitat Animal (CReSA IRTA-UAB), Campus de la Universitat Autònoma de Barcelona, Bellaterra, Spain; ^7^Department of Surgery, University of Pittsburgh School of Medicine, Pittsburgh, PA, United States; ^8^Department of Molecular Genetics and Biochemistry, University of Pittsburgh School of Medicine, Pittsburgh, PA, United States; ^9^Department of Medicine, University of Pittsburgh School of Medicine, Pittsburgh, PA, United States; ^10^Università Cattolica del Sacro Cuore, Rome, Italy

**Keywords:** haemophilus (Glaesserella) parasuis, molecular mimicry, rheumatoid arthritis, host-pathogen interaction, cross-reactivity

## Abstract

**Background:***Haemophilus parasuis* (*Hps*; now *Glaesserella parasuis*) is an infectious agent that causes severe arthritis in swines and shares sequence similarity with residues 261–273 of collagen type 2 (Coll_261−273_), a possible autoantigen in rheumatoid arthritis (RA).

**Objectives/methods:** We tested the presence of *Hps* sequencing 16S ribosomal RNA in crevicular fluid, synovial fluids, and tissues in patients with arthritis (RA and other peripheral arthritides) and in healthy controls. Moreover, we examined the cross-recognition of *Hps* by Coll_261−273_-specific T cells in HLA-DRB1^*^04^pos^ RA patients, by T-cell receptor (TCR) beta chain spectratyping and T-cell phenotyping.

**Results:***Hps* DNA was present in 57.4% of the tooth crevicular fluids of RA patients and in 31.6% of controls. Anti-*Hps* IgM and IgG titers were detectable and correlated with disease duration and the age of the patients. Peripheral blood mononuclear cells (PBMCs) were stimulated with *Hps* virulence-associated trimeric autotransporter peptide (VtaA10_755−766_), homologous to human Coll_261−273_ or co-cultured with live *Hps*. In both conditions, the expanded TCR repertoire overlapped with Coll_261−273_ and led to the production of IL-17.

**Discussion:** We show that the DNA of an infectious agent (*Hps*), not previously described as pathogen in humans, is present in most patients with RA and that an *Hps* peptide is able to activate T cells specific for Coll_261−273_, likely inducing or maintaining a molecular mimicry mechanism.

**Conclusion:** The cross-reactivity between VtaA10_755−766_ of a non-human infectious agent and human Coll_261−273_ suggests an involvement in the pathogenesis of RA. This mechanism appears emphasized in predisposed individuals, such as patients with shared epitope.

## Introduction

Rheumatoid arthritis (RA) is a chronic inflammatory disease of the joints that progressively destroys the cartilage and the subchondral bone (1, 2). Susceptibility to RA is associated with HLA-DRB1 locus (shared epitope) and antibodies against citrullinated peptide (ACPA) (3–5), revealing a strict pathogenic relationship between HLA class II-dependent immune response (4, 6, 7) and the determinant role of T cells along with B cells in this disease (8). Antibodies directed against citrullinated peptides (fibrinogen, vimentin, enolase, collagen type II, etc.) can develop years before the occurrence of the first symptoms (4, 9, 10), highlighting the role that a slow adaptive autoreactive immune response plays in driving disease occurrence.

One of the suspected autoantigens is the cartilage collagen type 2, a major component of the target in inflammation. We previously demonstrated that T cells characterized by specific T-cell receptor (TCR) public clonotypes expanded in response to collagen type 2 peptide 261–273 (Coll_261−273_) are specifically expressed by RA patients compared to controls, are present in the blood during the early active phase, disappear during remission, and reoccur during relapses (11). These clonotypes mostly use a restricted repertoire of variable beta (BV) chains and we demonstrated that definite sequences of the TCR interact with the autoantigen, most particularly the sequence CASS DTGS SGAN (12, 13).

The autoantigen Coll_261−273_ shares sequence similarities highly homologous to *Capnocytophaga spp*. (*Cg*) and *Streptococcus pyogenes* (*Spy*), but especially to *Haemophilus parasuis* (*Hps*; now *Glaesserella parasuis*); and since interesting associations emerged between different infectious agents, pathobionts or commensals, and RA (14), we tested in this study the hypothesis that one or more infectious agents could break down tolerance to self-components and may play a relevant role in the pathogenesis of RA, through molecular mimicry (15, 16).

Peptide 755–766 from the protein VtaA10 (VtaA10_755−766_) of *Hps* overlaps almost completely with Coll_261−273_, but to date, no reports have shown an involvement of *Hps* in human diseases. *Hps* is known to behave as a pathogen only in pigs (17, 18). Intriguingly, in this animal, *Hps* causes Glässer's disease, characterized by severe arthritis (plus meningitis, pericarditis, and polyserositis) with a poorly understood pathogenesis (19). Despite a higher incidence of RA in a large group of Canadian farmers being reported (20), to date, there are no other reports indicating pork meat consumption or farming as possible triggers for RA (21, 22).

The data we present here show that *Hps* DNA can be found in the gingival crevicular fluid of RA patients at a frequency higher than in healthy controls (HCs > 30 years old), not different from Young Healthy Controls (HCs <30 years old), in synovial fluid and tissues. Public T-cell clonotypes specifically reacting with the Coll_261−273_ peptide react with live *Hps*, cross-react with *Hps* VtaA10_755−766_ and with live VtaA10^+^ *Hps*, and produce IL-17 more often than IL-13 (11).

## Materials and Methods

### Patients

A total of 100 consecutive patients with active arthritis at the Division of Rheumatology of the Fondazione Policlinico Universitario “A. Gemelli” I.R.C.C.S. of Rome have been included in this study. The cohort is composed of 22 patients with undifferentiated peripheral inflammatory arthritis (UPIA), 47 RA patients [all satisfying the 2010 American College of Rheumatology (ACR) classification criteria] (23), and 31 patients with arthritides of different origins [ten with psoriatic arthritis (PsA), four with systemic lupus erythematosus (SLE), three with osteoarthritis (OA), three with juvenile idiopathic arthritis (JIA), two with Reiter's syndrome, two with polymyalgia rheumatica, and one each with chondrocalcinosis, eosinophilic fasciitis, gout, spondyloarthritis (SpA), villonodular synovitis, Sjogren's syndrome, and Still's disease]. Demographic and clinical characteristics are described in Table 1.

**Table 1:** Demographic, immunological, and clinical characteristics of patients with arthritides and healthy controls.

**Characteristics**	**RA**	**UPIA**	**Others**	***p***	**HS>30**	**HS <25**
*N*	47	22	31		38	131
Age, (years)	56.4 ± 15.7	42.4 ± 14.0	40.8 ± 17.0	** <0.001**	49.4 ± 8.0	22.7 ± 1.5
Sex, no female, (%)	39 (83.0)	15 (68.2)	17 (63)	0.28	15 (75)	79 (60.3)
Age Onset, mean ± SD	51.4 ± 16.3	39.5 ± 14.8	35.9 ± 18.2	** <0.001**	N/A	N/A
Disease Duration, (years)	5.0 ± 6.9	3.0 ± 4.0	4.9 ± 6.6	0.53	N/A	N/A
BMI, mean number ± SD	24.8 ± 5.1	24.3 ± 4.2	24.2 ± 3.9	0.98	N/A	N/A
Smokers, no (%)	13/42 (30.9)	7/20 (35.0)	6/27 (22.2)	0.32	Nd	Nd
Ex-smokers, no (%)	8/42 (19.1)	2/20 (10.0)	5/27(18.5)	0.65	Nd	Nd
Ab positive, no (%)	27 (57.4)	2 (9.1)	1 (3.2)	** <0.001**	Nd	Nd
ESR (mm/1 ^∧^ h)	54.1 ± 37.1	36.6 ± 36.3	39.0 ± 30.2	**0.04**	Nd	Nd
CRP (mg/l)	26.2 ± 33.7	22.9 ± 33.3	24.8 ± 40.6	0.5	Nd	Nd
DAS, score ± SD	3.5 ± 1.3	2.4 ± 0.7	N/A	N/A	N/A	N/A
TJ44, mean number ± SD	10.3 ± 9.9	2.9 ± 3.2	N/A	N/A	N/A	N/A
SJ44, mean number ± SD	7.7 ± 7.3	2.1 ± 1.4	N/A	N/A	N/A	N/A
HAQ, mean number ± SD	1.3 ± 0.9	0.6 ± 0.7	N/A	N/A	N/A	N/A

*Values are mean ± standard deviation unless otherwise indicated. Ab positivity on the basis of at least a positivity for ACPA (anti-citrullinated peptide antibodies) and/or RF (rheumatoid factor IgM and/or IgA); CRP, C-reactive protein; ESR, erythrocyte sedimentation rate; DAS, disease activity score; TJ44, tender joints; SJ44, swollen joints; HAQ, health assessment questionnaire (disability index); BMI, body Mass Index; Others, other arthritides; RA, rheumatoid arthritis; UPIA, undifferentiated peripheral inflammatory arthritis; Others arthritides: patients affected by arthritis with different other diagnosis: 10 psoriatic arthritis (PsA), 1 chondrocalcinosis, 1 Eosinophilic Fasciitis, 1 Gout, 4 systemic lupus erythematosus (SLE), 1 spondylo-arthritis (SpA), 2 Reiter syndromes, 2 Polymyalgia Rheumatica, 1 villonodular Synovitis, 1 Sjögren Syndrome, 1 Still's Disease, 3 Osteoarthritis (OA) and 3 Juvenile Idiopathic Arthritis (JIA). SD, standard deviation. Statistical analyses are performed as described in methods. Significant differences are highlighted in bold displaying the p-values after ANOVA analysis*.

Thirty-eight aged healthy donors (>30 years old) and 131 young healthy donors (21–25 years old) were included in the study also. The research follows the Declaration of Helsinki. The ethical approval for the study was obtained from the Università Cattolica del Sacro Cuore Ethical Committee. Informed written consent was obtained from all the patients and healthy donors.

The available samples from enrolled patients were as follows: (a) peripheral blood; (b) sub-gingival dental plaque as previously described (24); and in 37 patients, also (c) synovial tissues through an ultrasound-guided percutaneous needle synovial biopsy (14G Tru-cut Precisa 1410—Hospital Service) (25). Forty-seven RA [34 long-standing RA (LSRA) and 13 early RA (ERA)] patients fulfilling at least six of the ACR/European League Against Rheumatism (ACR/EULAR) criteria for RA were considered as the case population. All patients were typed for HLA-DRB1. Ten subjects were tested by Immunoscope technique (26) (primers are listed in Supplementary Table 1); they were all ERA and were being treated following a standardized strategy (treat to target) in an outpatient setting. RA patients with LSRA were excluded from this latter analysis to avoid pharmacological treatment diversity bias. Fifty out of 100 enrolled patients were tested for IgG and IgM antibodies specific for *Haemophilus parasuis* as described below.

### Presence of Bacterial DNA in Crevicular Fluids, Synovial Fluids, and Synovial Tissues

*Hps* DNA was detected by polymerase chain reaction (PCR) amplifying species-specific sequences on the 16S rRNA gene proved useful for detection of *Hps* in clinical samples (27) and following published protocols (28, 29). Similarly the presence of *Porphyromonas gingivalis* (*Pg*) (30), *S. pyogenes* (*Spy*) (31), and *Capnocytophaga* spp. (*Cg*) (32) DNA was detected following protocols from the literature. Primers are listed in Supplementary Table 1.

### Antibody Response—ELISA

The complete passenger domain of a representative VtaA from group 1 (VtaA9) and from group 2 (VtaA10) of the virulent Nagasaki strain, together with the stalk, connector (amino acids 589–929, which contain the collagen domains of the protein), and head fragments of VtaA10 were produced as recombinant proteins as previously described (33). The recombinant VtaAs and VtaA10 fragments were used to evaluate serum IgG and IgM using an in-house ELISA. High-binding plates were coated with 0.5 μg/well of each of the five proteins and were subsequently blocked with 5% skim milk. The sera were diluted 1:100 for IgG and IgM detection. IgG antibodies were detected with a horseradish peroxidase (HRP)-conjugated protein A and IgM antibodies with an HRP-conjugated anti-human IgM. Positive reactions were observed with the HRP substrate 3,3′,5,5-tetramethylbenzidine. Similarly, an *Hps* bacterin was used in ELISA to measure IgG and IgM using 1:500 and 1:100 dilution of the sera, respectively.

To determine the presence and the concentrations of IgG against type II collagen in the sera of patients, the Human/Monkey Anti-Human type II Collagen IgG Antibody Assay Kit was used following manufacturer's instructions (Chondrex, Woodinville, WA, USA). For each sample, patients were scored positive if the optical density (OD) values in the ELISA of anti-CII antibody concentration exceeded the mean OD value by more than 3 standard deviations of controls. The data were analyzed by linear regression analysis, assuming a Poisson distribution. Frequencies were calculated from the slope of the curves. Levels >95th percentile of controls (109 AU/mL) were considered positive, as reported by Manivel et al. (34).

### Sequence Analysis and Sequence Similarity Searches

The genomic DNA samples positive for the *Hps* detection above described (26 patients and 31 HCs) were sequenced for 16S gene, using BigDYE 3.1 and a Genetic Analyzer 3130 (Life Technologies, Carlsbad, CA, USA).

For homology searches, we ran BLASTP (protein-protein BLAST) against the Bacteria (taxid 2) database of the National Center for Biotechnology Information (NCBI) non-redundant protein (nr) GenBank CDS translations+ PDB+ SwissProt+ PIR+ PRF excluding environmental samples from whole-genome sequencing (WGS) projects (146,243,064 sequences; 53,601,822,492 total letters). The 13aa sequence AGFKGEQGPKGEP search parameters were adjusted to search for a short input sequence (35).

CD4^+^ restricted Coll_261−273_ T-cell epitope AGFKGEQGPKGEP was blasted against non-redundant protein sequences of bacteria (taxid:2). The cutoff was determined by the identities, positives, and presence of gaps.

### Cell Cultures, Immunoscope, and HLA-DRB1 Genotyping

Peripheral blood mononuclear cells (PBMCs) were collected from 13 ERA patients in different phases of the disease (Supplementary Table 2) and cultured as described in our previous work (8, 11) in four different conditions: (i) unstimulated or in the presence of: (ii) stimulation with Coll_261−273_ (AGFKGEQGPKGEP) at a concentration of 20 mg/ml; (iii) VtaA10_755−766_ (AGPKGEQPKGE) at the same concentration; or (iv) in co-culture with 10^6^ CFU/well of *Hps* ATCC® 19417™. In addition, PBMCs from two ERA patients [one RA patient at his first remission of disease and the second with moderate disease activity score (DAS)], after *in vitro* stimulation with Coll_261−273_ or co-culture with *Hps*, were enriched on the basis of the IL-17 or IL-13 secretion by MACS® secretion assay (Miltenyi Biotec, North Rhine-Westphalia, Germany) (36).

In order to perform the analysis of the TCR repertoire, we used the Immunoscope technique, a reverse transcriptase–PCR (RT-PCR)-based method, which subdivides any bulk population of T cells into approximately 2,400 categories based upon different Vβ-Jβ gene combinations (37, 38). DNA sample for Immunoscope was first amplified with forward and reverse primers to the variable and constant region segments, respectively (Supplementary Table 1). Next, fluorescent primers to each of the joining segments were used for “run-off” reactions. The Vβ, Cβ, and Jβ primers are listed in Supplementary Table 1 (8, 11, 39). The “run-off” products were then analyzed using a Genetic Analyzer and according to nucleotide length fractioned in a spectrum with different peaks, each of which represents a polyclonal T-cell population sharing the same BV–junctional beta (BJ) rearrangement and the same length of CDR3, but not the same sequence. A Gaussian distribution of CDR3 lengths was observed in the spectra in unstimulated samples. In contrast, clonal expansions were observed as perturbations of the Gaussian or as the presence of single peak, which represented an oligoclonal or monoclonal T-cell population, respectively, not only sharing the length of the CDR3 and the BV–BJ rearrangement but also having a conserved CDR3 sequence. Sequencing of public TCRs studied in this paper was performed and published in previous works (11).

In addition, PBMCs from two ERA patients (both chosen as proof of concept), after *in vitro* stimulation with Coll_261−273_ or co-culture with *Hps*, were enriched on the basis of the IL-17 or IL-13 secretion by MACS® secretion assay (Miltenyi). The presence of specific TCRs was assessed on T cells that were allowed to proliferate for 3 days in response to the peptide antigens or to the co-culture.

HLA-DRB1 genotyping was performed using the INNO-LiPA HLA-DRB1 plus kit (Fujirebio, Tokyo, Japan) following manufacturer's instructions.

### Statistical Analysis

Data were analyzed using SPSS Statistics 22.0 (IBM, Armonk, NY, USA) and Prism software 8.0 (GraphPad, San Diego, CA, USA). Categorical and quantitative variables were recorded as frequencies, percentage, and mean ± standard deviation (SD), as appropriate. The non-parametric Mann–Whitney *U*-test was used to compare the continuous variables. Categorical variables were analyzed using χ^2^ test or Fisher's exact test, as appropriate. Spearman's rank correlation was used to evaluate the relationship between IgM bacterin, IgM VtaA9, and IgM VtaA10 levels and demographic clinical parameters. *p* < 0.05 were considered statistically significant.

## Results

### *Hps* Is Detected at the Highest Frequency in Young Healthy Patients, at the Lowest Frequency in Older Healthy Controls, and at Intermediate Frequency in Rheumatoid Arthritis Patients

Clinical characteristics of RA patients, UPIA patients, and HCs enrolled in this study are detailed in Table 1. RA patients had a high disease activity and a high disability at the moment of the biopsy [mean DAS 44 3.5 ± 1.4 (normal values <1.6), mean HAQ-DI 1.3 ± 0.90 (normal values < 0.5)]. Disease activity and disability were higher in RA patients than in patients affected by UPIA.

A total of 27 (57.4%) RA patients, two (9.1%) with UPIA and one (3.2%) with other arthritides (one out the 10 affected by PsA), were positive for at least one tested autoantibody [ACPA, IgM rheumatoid factor (RF), and IgA RF]. The presence of joint erosions was significantly higher in RA patients compared with UPIA (63.3 vs. 25.0%, *p* = 0.01, data not shown) and compared with patients affected by other arthritides (22.7%, all PsA, *p* < 0.01 vs. RA patients, data not shown). ERA patients had a lower prevalence of erosions than patients with an LSRA (22.2 vs. 81.0%, *p* < 0.01, data not shown). Groups were comparable for disease-modifying antirheumatic drugs (DMARDs) and steroid therapy, although RA patients were more frequently receiving biologic therapy (RA vs. other arthritides: *p* < 0.01, data not shown).

**Figure 1:**
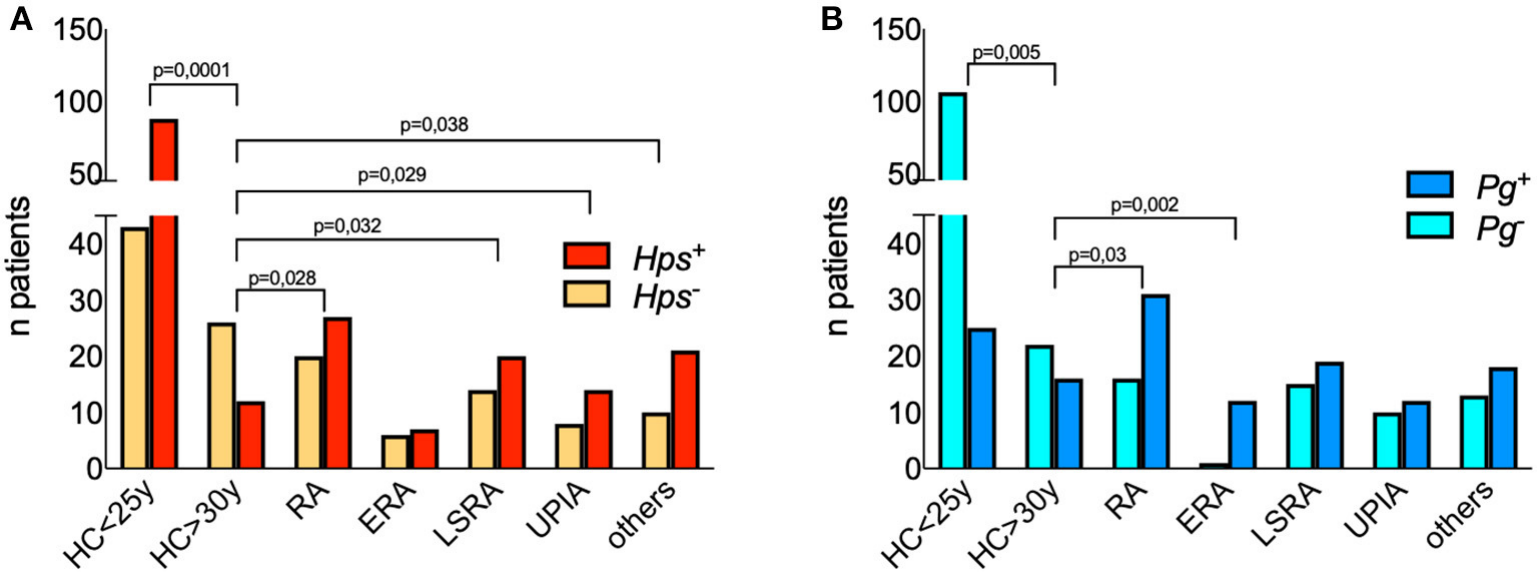
Detection of 16S DNA for *Hps* and *Pg* in the crevicular fluid of patients and controls. Each bar represents the numbers of patients and healthy controls positive for Pg **(A)** and Hps **(B)**. Differences between proportions have been calculated with two-sided Fisher's Exact test and significant p values are displayed among groups. *Hps* DNA was detected by polymerase chain reaction (PCR) amplifying species-specific sequences on the 16S rRNA gene proved useful for detection of *Hps* in clinical samples 21. The following primer's pairs were used in a nested-PCR. Outer primers: Hps16S-forw 5′AGAGTTTGATCATGGCTCAGA3′ and *Hps*16S-rev 5′AGTCATGAATCATACCGTGGTA3′ 22; inner primers: *Hps*-forw 5′GTG ATG AGG AAG GGT GGT GT3′ and *Hps*-rev 5′GGC TTC GTC ACC CTC TGT3′ 23. The predicted size of the final PCR product was 821 base pairs (bp). In order to detect the presence of Pg DNA were used 5′-AGG CAG CTT GCC ATA CTG CG-3′ and 5′-ACT GTT AGC AAC TAC CGA TGT-3′ oligonucleotides, for a 404 bp amplicon derived from the 16S rRNA.

We first assessed the presence of DNA from *Hps, Pg, Cg*, and *Spy* in the crevicular fluids of 100 patients (Table 1). Results are summarized in Figures 1A,B, Supplementary Figure 1A, and Supplementary Table 2.

*Hps* DNA was found in the crevicular fluids of 27 RA patients (out of 47, 57.4%). In two ERA patients (out of 13 examined) it was possible to find *Hps* DNA in the synovial tissues and fluids. The frequency of positivity in the crevicular fluid of patients suffering from other arthritides was similar to that observed in RA (Figure 1A).

Given the loss of tolerance to self-antigens, which likely happens several years before onset of autoimmune diseases such as RA, our first step was to dissect the ability of *Hps* to infect human subjects in two groups of healthy individuals defined as “young” (<25 years) or “old” (>30 years) controls. The age of this latter cohort was comparable with that of the group of RA patients studied in this work. We found that *Hps* was present in 87 out of 131 medical students, males and females, between 21 and 25 years (66.4%), while only 12 out 38 (31.6%) crevicular fluids still tested positive for *Hps* DNA (*p* < 0.0001, two-sided Fisher's exact test) in older HCs (>30 years). The comparison of *Hps* positivity rates among age-matched controls and the groups of patients revealed significant differences (HCs >30 vs. RA, LSRA, UPIA, and other arthritides showed *p*-values of 0.028, 0.032, 0.029, and 0.038 respectively, two-sided Fisher's exact test), except for ERA patients (*p* = 0.2; Figure 1A).

We found that DNA of *Pg* was present in both young and old controls (19.1 and 42.1%, respectively), while LSRA, UPIA, and patients suffering from arthritis of other origin had a frequency of positive results between 50 and 60% (Supplementary Figure 1A). As previously described (30), ERA patients showed a high frequency of positivity for *Pg* (90%), significantly more than HCs >30 years (*p* = 0.002; Figure 1B).

DNA specific for *Cg* was present in all groups in the majority of samples, while DNA of *Spy* was frequently positive only in the LSRA group (Supplementary Figure 1A). We found that only 10% of the patients had positive anti type 2 collagen autoantibodies; clustering RA patients based on the presence of *Hps* and *Pg* DNA (Figures 1A,B) and of antibodies specific for type 2 collagen (10% of the cohort, Supplementary Figure 1B), there were no significant differences in terms of disease severity, age at onset, and seropositivity, although *Hps*+ RA patients showed a trend of relatively low titers of ACPA, RF-IgM, and IgA.

To confirm the positivity for *Hps*, we sequenced the DNA obtained from crevicular fluid of 26 RA patients (among the 27 *Hps* positive, one sample from RA patient was missing due to insufficiency for sequencing experiment) and 31 young HCs positive for *Hps*. We obtained a sequence identity of the 16S rRNA gene of *Hps* >95% as compared with the one reported in the gene database in the NIH Public Library (11), in 10 out of 26 patients and 13/31 young controls. In one case, we found an identity of 100%. In most of the cases, the missing identity of our sequence was concentrated in few segments and was due to small nucleotide substitution as summarized in Supplementary Table 3 and specifically shown in Supplementary Table 4 and Supplementary Figure 2.

### The Antibody Response to *Hps* in Rheumatoid Arthritis and Undifferentiated Peripheral Inflammatory Arthritis Patients

Microbe-specific antibodies to date represent a marker of the individual history of infections (40). We therefore measured the presence of IgM and IgG (for recent/chronic colonization) specific for three *Hps* antigens, bacterin, VtaA9, and VtaA10. IgM and IgG specific for VtaA9 and VtaA10 titers did not differ among patients with RA or other arthritides (Supplementary Figure 3). Within the RA group, the mean levels of anti-bacterin IgG were consistently higher, though not statistically different, in the subgroup of LSRA patients than in ERA patients (1.62 ± 0.52 vs. 1.17 ± 0.41, *p* = 0.53, Mann–Whitney *U*-test). No differences were observed between HLA-DR1/HLA-DR4 positive or negative RA patients (Supplementary Figure 4).

**Figure 2:**
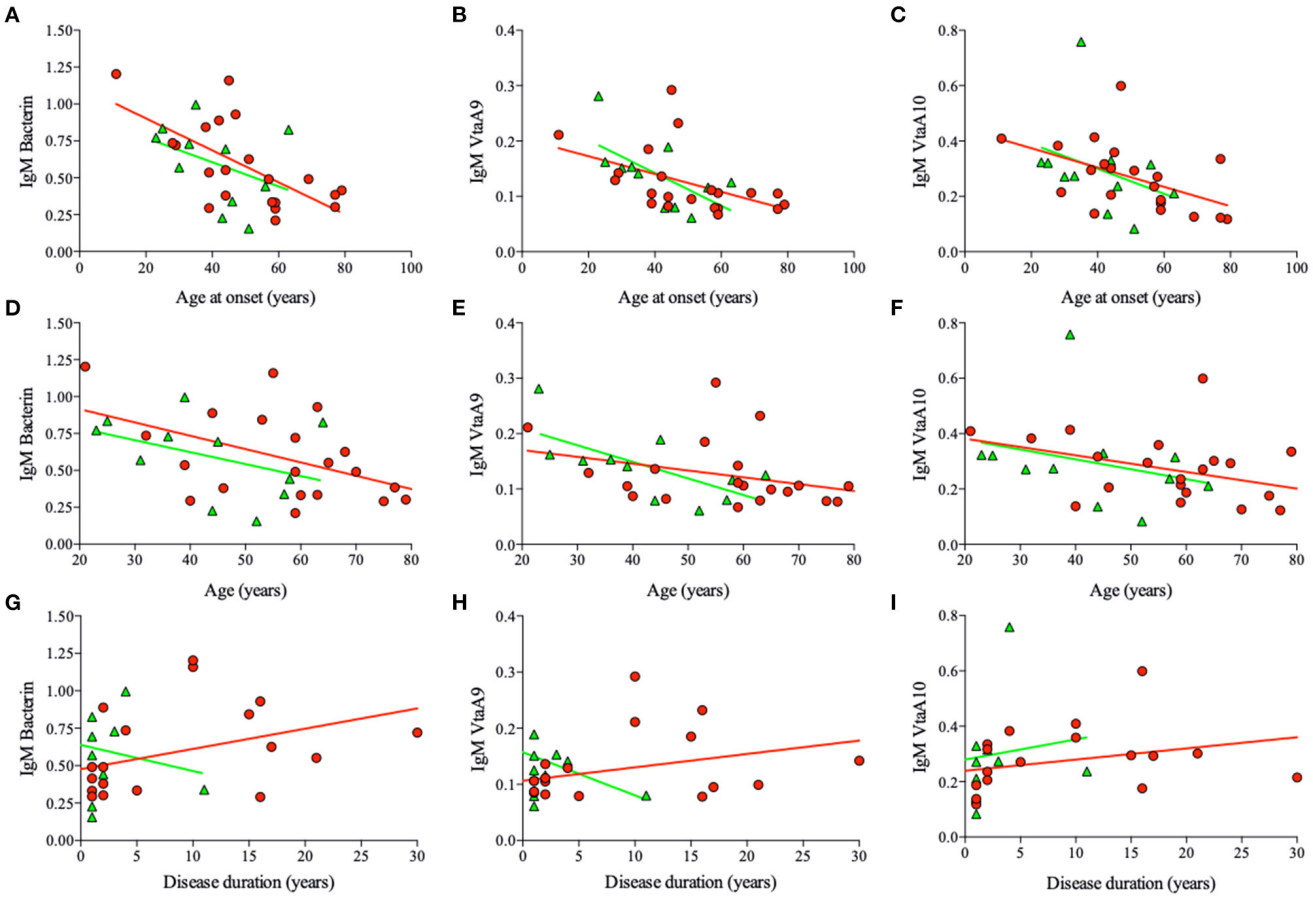
*Hps* specific IgM titers and main correlations. Correlation between IgM titers specific for Bacterin, VtaA9, and VtaA10, specific for *Hps*, and Age at the onset, Age, and disease duration from the sera of the RA (red circles) and UPIA (green triangles) patients. In RA IgM titers inversely correlate with age at onset (**A**, bacterin, *r* = −0.55, *p* = 0.01. **B**, VtaA9, *r* = −0.41, *p* = 0.05. **C**, VtaA10, *r* = −0.40, *p* = 0.02), age (**D**, ns; *p* = 0.02. **E**, ns. **F**, ns), and directly correlate with the disease duration (**G**, bacterin, *r* = 0.38, *p* = 0.07. **H**, ns. **I**, ns.). In UPIA patients IgM titers inversely correlate with the age at the onset (**B**, VtaA9, *r* = −0.60, *p* = 0.09) and the age (**E**, VtaA9, *r* = −0.69, *p* = 0.04).

In RA patients, the amount of IgM specific for bacterin, VtaA9, and VtaA10 unexpectedly increased with disease duration; in UPIA patients, on the contrary, the amount of IgM specific for bacterin and VtaA9 decreased with disease duration. Thus, reinfections by *Hps* might play an active immunologic role specifically in RA (Figures 2G–I).

Other correlations (Spearman's rank correlations) were observed between age at onset and specific anti-*Hps* IgM. We found inverse correlations between age at onset of the RA patients and antibody titers, such as bacterin-specific IgM (*R* = −0.55, *p* = 0.01), VtaA9-specific IgM (*R* = −0.41, *p* = 0.05), and close to significance for VtaA10-specific IgM (*R* = −0.40, *p* = 0.06; Figures 2A–C, respectively; Spearman's rank correlations). For VtaA9 IgM/IgG ratio (*R* = −0.4, *p* = 0.06, Spearman's rank correlation), the inverse correlation was not significant if considered based on the age of the patients (Figures 2D–F).

Finally, *Hps*-specific IgG and IgM levels did not associate to age or age at disease onset in UPIA patients (Figure 2 and Supplementary Figure 5).

When examining the levels of IgM specific for VtaA10 as a function of the age in non-RA patients, we observed that the highest ones were found in subjects in their third decade of life (Supplementary Figure 6). This observation indicated that colonization and sero-conversion are more likely to occur during the second/third decade of life, in most normal subjects. This observation was consistent with the above-reported results regarding the high frequency of detection of *Hps* DNA in the cohort of young healthy subjects. Taken together, these collected data indicate that infection by *Hps* appears to be a common event.

### A Large Fraction of T-Cell Receptors Specific for huColl_261–273_ Cross-Recognize VtaA10_755–766_ Also From Live Hps and Produce IL-17A

In a search for epitopes of microbial origin potentially able to sustain mimicry with human type 2 collagen, we found that peptide 755–766 from VtaA10 protein of *Hps* (a protein found only in strains that are arthritogenic in swine) almost completely overlapped with peptide 261–273 of human type 2 collagen that comprises the dominant epitope for this protein in the HLA-DRB1^*^04 and 01. The only different residue between the two sequences was residue Phe263 in collagen for residue Pro757 in VtaA10. This substitution affected the huColl_261−273_ peptide anchor residue for DRB1^*^04 binding pocket 1, according to our previous work (12).

By using the Immunoscope technique, we previously described several TCR beta-chains frequently used by huColl_261−273_-specific T cells in DRB1^*^04 ERA patients (8, 11). We therefore examined if these public T cells recognizing huColl_261−273_ could also be stimulated by VtaA10_755−766_ and by live *Hps* producing this protein, in a limited group of ERA patients (*n* = 13, 7 of which were DRB1^*^04^+^). The clinical and demographic characteristics of these patients are described in Supplementary Table 5. The presence and radiosensitivity index (RSI) of T cells carrying the public TCRs were consistent with those described in our previous papers (8, 11).

PBMCs from ERA patients were cultured without antigen (background) or in the presence of Coll_261−273_, or of VtaA10_755−766_ or with 10^6^ CFU of *Hps* as previously described (41). Immunoscope analysis was then performed on PBMCs, and on enriched IL-13- or IL-17A-producing T cells upon antigen stimulation, as previously described (11).

**Figure 3:**
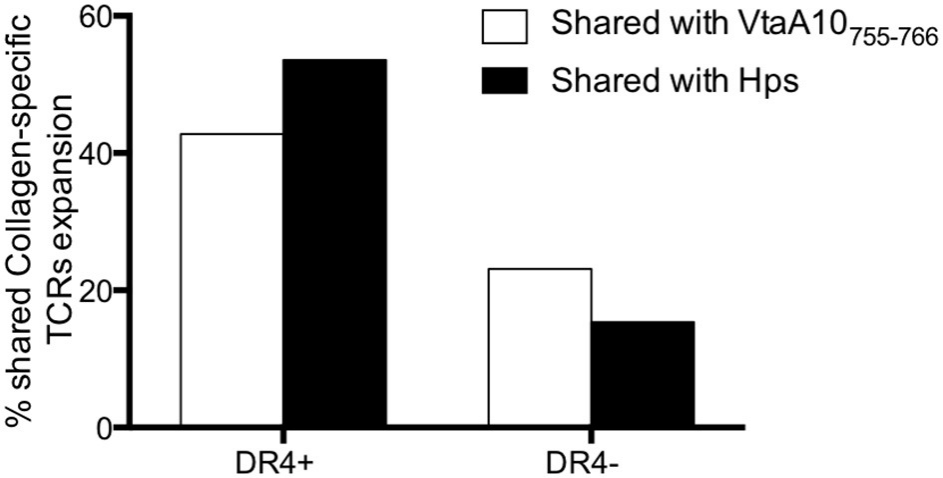
Collagen_261−273_-specific, shared TCRs expand in response to VtaA_755−766_ and to live VtaA10^+^ Hps. Comparison of the results obtained by Immunoscope analysis of TCR repertoire of the PBMC from 13 ERA patients of whom demographic, immunological, and clinical characteristics are displayed in Supplementary Table 4. PBMC were cultured in the following conditions: without antigen (negative control), with Collagen_261−273_ (AGFKGEQPKGE) or VtaA_755−766_ peptide (AGPKGEQPKGE) or co-cultured with *Haemophilus parasuis* ATCC® 19417^TM^. Once obtained the TCR repertoire for each condition (260 Vbeta-Jbeta rearrangements for each stimulation, data not shown) we compared the VtaA_755−766_-specific and the live Hps-specific repertoires with the Collagen_261−273_-specific one, all normalized for the unstimulated cells repertoire. The white bars show the overlapping of TCR repertoire after VtaA_755−766_ stimulation with the one after Collagen stimulation, both normalized on the TCRs from unstimulated PBMC. With the black bars the TCR repertoires are displayed comparing Hps co-culture with Collagen stimulation. In DR4^+^ patients the overlap concerns a large part of the T cells repertoires, much more than DR4 negative ERA patients.

The stimulation with VtaA10_755−766_ was able to induce proliferation of 12/28 (42.8%) T cells carrying the same TCRs of those stimulated with Coll_261−273_, at the same peptide concentration, in DRB1^*^04^pos^ RA patients (in only six out 26, TCRs, 23.1%, in DRB1^*^04^neg^ patients). The actual availability of an epitope also depends on the ability of APCs to generate it from the protein and on the ability of bacteria to produce it in sufficient amounts. Thus, it is relevant that collagen-specific TCRs were stimulated by live VtaA10^+^ *Hps* even more frequently (15/28, 53.6%) in DRB1^*^04^pos^ RA patients than VtaA10_755−766_, as shown in Figure 3 and detailed in Supplementary Table 2. No clonotype expansion was detectable in the other arthritides.

**Figure 4:**
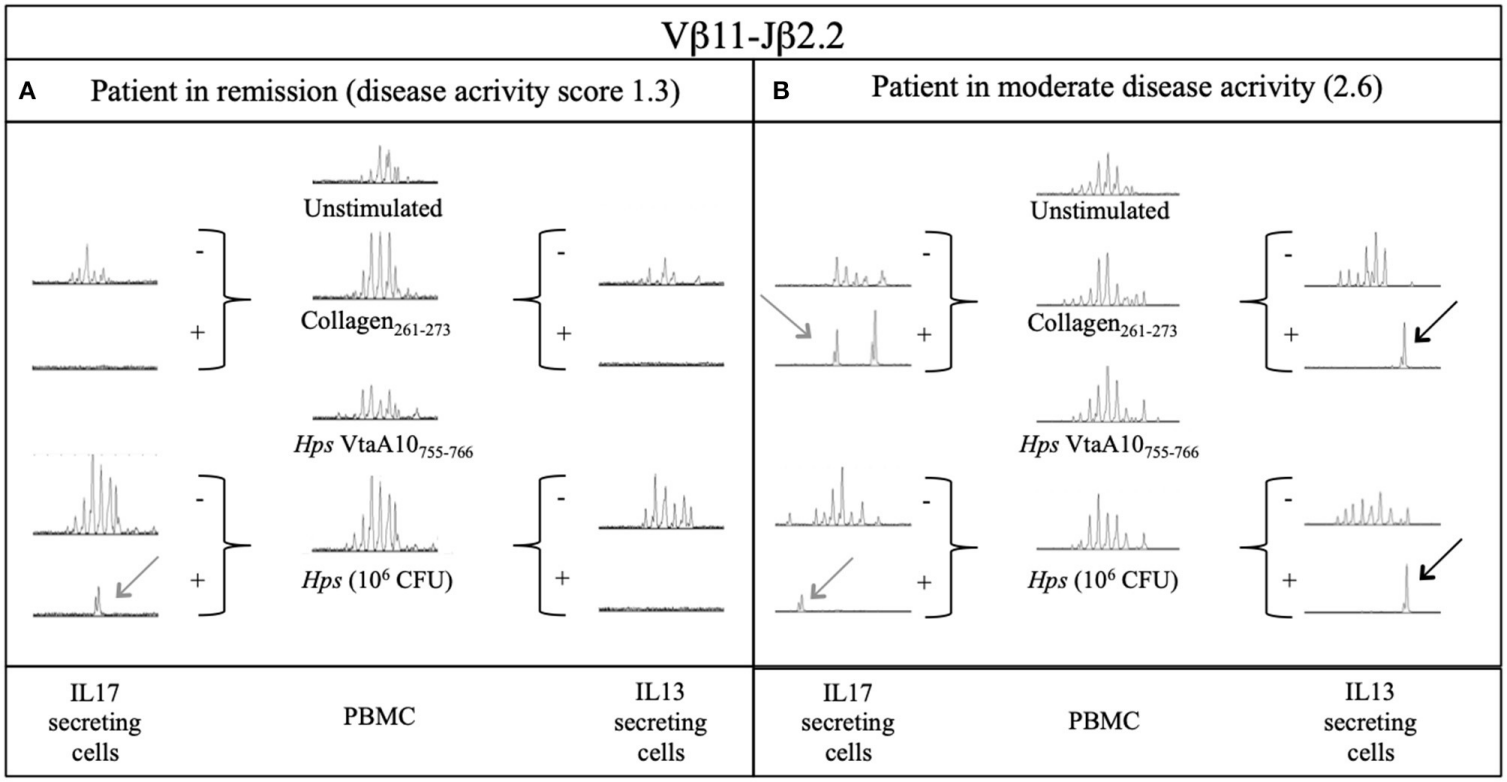
Secretion of IL17A and IL13 by Vβ11-Jβ2.2 rearrangement from two DR4+ ERA patients after stimulation with Collagen and live Hps. PBMC samples and sorted IL17 and IL13 secreting T cells from 2 ERA patients with different disease activity (DAS): **(A)** one in remission and **(B)** the other during active phase of disease were compared through Immunoscope analysis. The presence of an antigen-specific expansion is displayed with a perturbation of the gaussian or the presence of a single peak where possible the comparison could be done through the rate between the area of the same length peaks of two different samples (RSI, rate stimulation index). Hps, Haemophilus (Glaesserella) Parasuis; CFU, Colony Forming Unit; IL17, Interleukin 17; IL13, Interleukin 13; VtaA, virulence-associated trimeric autotransporter.

T cells obtained from two samples were also tested for their ability to secrete IL-17A or IL-13 in response to stimulation with Coll_261−273_ or live *Hps*. IL-17-secreting cells were detected in both samples, but it appeared that T cells from the patient in remission of disease (DAS 1.3) required bacterial (*Hps*)-derived moieties to be induced to produce IL-17A (Figure 4A). Coll_261−273_-specific TRBV25^+^ IL-13^+^ T cells were detected in only one of the two ERA patients (Figure 4B). Regarding the other collagen-specific TCR rearrangements that we described in our study, T cells carrying those TCRs appeared not to secrete IL-17 or IL-13 under any type of antigen stimulation (Supplementary Figures 7–10).

## Discussion

Here, we present data showing that an infectious agent not previously described as pathogenic in humans, *H. parasuis*, now *G. parasuis*, can trigger a pro-inflammatory response through molecular mimicry in RA, enhancing IL-17 production similarly to Coll_261−273_ peptide in shared epitope-positive patients.

In swine, *Hps* infection can induce severe arthritis, thus fulfilling the need to produce a similar disease in an animal model (17, 18). Considering that some VtaAs are associated with pathogenicity, an outcome leading to arthritis will depend on the strain of *Hps* and the genetic background of infected individuals in both pigs and humans.

We found that *Hps* frequently colonized crevicular fluid of healthy subjects in their twenties. At an older age, however, the detection rate of *Hps* DNA in the crevicular fluid of HCs decreased, suggesting that a protective immune response may have risen. Patients from all groups showed a high frequency of positivity for *Hps* DNA in their crevicular fluid. However, in light of a recent report identifying a close relative of *Hps* in humans, *Haemophilus massiliensis* (42) and with our data obtained with 16S rRNA gene sequence, we cannot completely rule out that this close relative of *Hps* might alternatively be present in some patients.

Comparing RA patients and HCs, we found no difference in the frequency of *Cg* and *Spy*, whereas only *Hps* and *Pg* colonize the oral mucosa more frequently, supporting the hypothesis that the immunologic effect of an early first infection is associated with rounds of re-stimulation with new infections.

*Hps* is able to stimulate autoreactive T cells specific for Coll_261−273_ peptide, inducing IL-17A production in an HLA-restricted manner. This agent may play a role as an inducer of a self-reactivity through molecular mimicry. Molecular mimicry has been considered one of the mechanisms that initiate and maintain autoimmunity (15, 43).

The group of T cells studied in this work meets the criteria for pathogenicity. Previously, we demonstrated that T cells specific for the autoantigenic Coll_261−273_ peptide are present in the blood during the active phases of RA disappear during the remission phases and reoccur at relapses (8). We have shown that these (auto)reactive T cells use a restricted repertoire of TCRs reproducibly committed in most patients, against autoantigen. This TCR selection occurs in a DRB1-restricted manner, being associated with those HLA alleles that are the strongest genetic risk factors for RA, not in other arthritides. Finally, we have shown that these TCRs (and, by extension, T cells) are found in the synovial fluids of RA patients at onset of the disease. Nevertheless, autoreactive T cells are present also in some DR4–patients, where, although with a different conformation and a different presentation, selected and expanded T-cell clones could cross-react to human self-antigen(s).

Several candidates have been proposed as disease-promoting microbes in RA, including viruses (44–48), mycobacteria (49–51), and other bacteria (14, 52–56). Thus far, however, none have been found as a candidate that could satisfy the criteria to be the culprit of RA.

To date (57, 58), two bacteria, both linked to periodontitis, have raised the greatest attention as being involved in RA pathogenesis: (i) *Pg*, because of its property of using peptidyl arginine-deiminase (PAD) to citrullinated proteins that become antigenic (30); (ii) *Aggregatibacter actinomycetemcomitans*, which may mimic membranolytic pathways by dysregulating the activation of citrullinating enzymes in the host neutrophils and trigger citrullination of autoantigen in RA joint(s) (14, 59).

We report here that a third infectious agent, *Hps*, carrying VtaA, a family of outer membrane proteins involved in the virulence process (60), contains an amino acidic sequence similar to the most immunogenic sequence of Coll_261−273_. *Hps* triggers the same T cells activated by the self-Coll_261−273_ and involved in disease pathogenesis. These autoreactive T cells produce IL-17A more often than IL-13 when stimulated by live *Hps*, contributing to the synthesis of the cytokine most linked to the acute phase of RA (61, 62). During disease, *Hps* persists in driving an active immune response, as witnessed by the increased level of *Hps*-specific IgM with disease duration.

*Hps* DNA was found in two synovial tissues, only from RA patients. This observation indicates differences in the pathogenesis of different arthritides. The acute presentation of RA can be the result of an “individually determined” autoimmune reaction, in which *Hps* may act as a (repeated) trigger of autoreactive response, or by modifying trafficking (63, 64) and secretory properties, of self-collagen-specific T cells, as illustrated in Figure 4B. However, in at least some cases, the acute presentation of RA may also be the result of an allo-(*Hps*)-specific immune response toward chronic or relapsing joint colonization, similar to what has been shown in other models (41).

The data reported in the present study suggest that VtaA10^+^ *Hps* colonization may play a role in the pathogenesis of RA, where reinfections or persistent infections appear to maintain an immune-driven ability. If this hypothesis is confirmed, a vaccination strategy using VtaA10- *Hps* in the first decade of life and/or an accurate prevention of re-infections through (e.g.), antibiotic clearance could open up new strategies for preventing or improving the outcome of RA. Certainly, since only some RA has been colonized, other agents may be involved (65).

In conclusion, *Hps*-induced molecular mimicry appears to be involved in the pathogenesis of RA. The pathogenicity of *Hps* in the animal model, the ability of the *Hps* epitope VtaA10_755−766_ to stimulate the same T-cell clonotypes that react to Coll_261−273_, the presence of *Hps* DNA in the crevicular fluid of most RA patients (and in the synovial tissues and fluids), and the presence of both IgM and IgG antibodies to the collagen domains of VtaA10 prove Witebsky's revised criteria for autoimmune diseases (66). Prospective studies in normal subjects carrying the DRB1^*^04 allele and positive for *Hps* should clarify how often and when subjects would develop RA. The possibility that an infectious agent could be adequately controlled by antibiotics or by vaccination might really change the future of RA, as demonstrated in previous combination studies (67, 68).

Further *in vivo* models might help to better understand whether *Hps* infection alone or in combination with *Pg* may actually lead to more arthritis, and studies in mice humanized for collagen II, CD4, and HLADRB1^*^04 will be needed to determine whether *Hps* infection can lead to rheumatoid-like arthritis (69).

## Data Availability Statement

The datasets presented in this study can be found in online repositories. The names of the repository/repositories and accession number(s) can be found at: GenBank MW657623 to MW657637.

## Ethics Statement

The studies involving human participants were reviewed and approved by Università Cattolica del Sacro Cuore. The patients/participants provided their written informed consent to participate in this study.

## Author Contributions

GD, PC, CD, MT, CC, SM, LP, SA, FR, and IP contributed to Immunoscope analysis and *Hps* sequencing. VA performed ELISA. BT, EG, and GD performed statistical analysis. PC, SM, CD, and BT performed and validated PCRs. GD, GF, FR, VA, MT, AG, PC, BT, and EG wrote the rationale, analyzed the data, and wrote and revised the manuscript. All authors contributed to the article and approved the submitted version.

## Conflict of Interest

The authors declare that the research was conducted in the absence of any commercial or financial relationships that could be construed as a potential conflict of interest.

## Publisher's Note

All claims expressed in this article are solely those of the authors and do not necessarily represent those of their affiliated organizations, or those of the publisher, the editors and the reviewers. Any product that may be evaluated in this article, or claim that may be made by its manufacturer, is not guaranteed or endorsed by the publisher.

## Abbreviations

*Hps*: *Haemophilus parasuis*
*Pg*: *Porphyromonas gingivalis*
*Cg*: *Capnocytophaga spp*.
*Spy*: *Streptococcus pyogenes*
Coll_261−273_: peptide 261–273 of human type II collagen
VtaA: virulence-associated trimeric autotransporter
PBMCs: peripheral blood mononuclear cells
TCR: T cell receptor
BV or vβ: variable region of the beta chain of T cell receptor
BJ or jβ: junctional region of the beta chain of T cell receptor
BC or cβ: constant region of the beta chain of T cell receptor
HLA: histocompatibility leucocyte antigen
RA: rheumatoid arthritis
UPIA: undifferentiated peripheral inflammatory arthritis
ERA: early rheumatoid arthritis
LSRA: long-standing rheumatoid arthritis
HC: healthy control
PsA: psoriatic arthritis
SpA: spondyloarthritis
JIA: juvenile idiopathic arthritis
DAS: disease activity score
HAQ: health assessment questionnaire
RF: rheumatoid factor
ACPA: anti-citrullinated protein antibodies
DMARDs: disease-modifying antirheumatic drugs.
