# Supplementary material for: Haemophilus parasuis (Glaesserella parasuis) as a Potential Driver of Molecular Mimicry and Inflammation in Rheumatoid Arthritis
Source: Front Med (Lausanne). 2021 Aug 17;8:671018. doi: 10.3389/fmed.2021.671018 (PMC8415917; doi:10.3389/fmed.2021.671018)
Supplement: Supplementary file 1 [file Table_1.docx]

|  | Primers name | Sequence 5’-3’ |  |
| --- | --- | --- | --- |
| *Haemophilus Parasuis*  Outer primers | *Hps*16S-forw | AGAGTTTGATCATGGCTCAGA | [^27-29^](#_ENREF_24) |
|  | *Hps*16S-rev | AGTCATGAATCATACCGTGGTA |  |
| *Haemophilus Parasuis*  Inner primers | *Hps*-forw | GTGATGAGGAAGGGTGGTGT |  |
|  | *Hps*-rev | GGCTTCGTCACCCTCTGT |  |
| *Porphiromonas Gingivalis* | *Pg*-forw | AGGCAGCTTGCCATACTGCG | ^30^ |
|  | *Pg*-rev | ACTGTTAGCAACTACCGATGT |  |
| *Streptococcus Pyogenes* | *Spy*1258-forw | AAAGACCGCCTTAACCACCT | ^31^ |
|  | *Spy*1258-rev | TGGCAAGGTAAACTTCTAAAGCA |  |
| *Capnocytophaga spp.* | *Cg*27-forw | GAGTTTGATCMTGGCTCAG | ^32^ |
|  | *Cg*1492-rev | TACGGYTACCTTGTTACGACTT |  |
| **T cell Receptor Beta Variable region (TRBV) / alias namesa** | **TRBV9/Hvβ1** | CCGCACAACAGTTCCCTGACTTGC | ^8, 11^ |
|  | **TRBV28/Hvβ3** | CGCTTCCCCTGATTCTGGAGTCC |  |
|  | **TRBV29/Hvβ4** | TTCCCATCAGCCGCCCAAACCTAA |  |
|  | **TRBV5 /Hvβ5** | GATCAAAACGAGAGGACAGC |  |
|  | **TRBV 7.2,7.3/Hvβ6a** | CATCCAATTTCAGGTCATACTG |  |
|  | **Hvβ6b1** | CAGGGCCAGAGTTTCTGAC |  |
|  | **TRBV7.4/Hvβ6b2** | CAGGGCTCAGAGGTTCTGAC |  |
|  | **TRBV4/Hvβ7** | CCTGAATGCCCCAACAGCTCT |  |
|  | **TRBV12/Hvβ8** | GGTACAGACAGACCATGATGC |  |
|  | **TRBV3/Hvβ9** | TTCCCTGGAGCTTGGTGACTCTGC |  |
|  | **TRBV21/Hvβ10** | CCACGGAGTCAGGGGACACAGCAC |  |
|  | **TRBV25/Hvβ11** | GTCAACAGTCTCCAGAATAAGG |  |
|  | **TRBV10/Hvβ12** | TCCACCTCACTCTGGAGTC |  |
|  | **TRBV6/Hvβ13a** | GGTATCGACAAGACCCAGGCA |  |
|  | **TRBV6.4/Hvβ13b** | AGGCTCATCCATTATTCAAATAC |  |
|  | **TRBV27/Hvβ14** | GGGCTGGGCTTAAGGCAGATCTAC |  |
|  | **TRBV24/Hvβ15** | CAGGCACAGGCTAAATTCTCCCTG |  |
|  | **TRBV14/Hvβ16** | GCCTGCAGAACTGGAGGATTCTGG |  |
|  | **TRBV19/Hvβ17** | TCCTCTACATGTGACATCGGCCCA |  |
|  | **TRBV18/Hvβ18** | CTGCTGAATTTCCCAAAGAGGGCC |  |
|  | **TRBV23/Hvβ19** | TCCCTCCACTGTGACATCGGCCCA |  |
|  | **TRBV30/Hvβ20** | TGCCCCAGAATCTCTCAGCCTCCA |  |
|  | **TRBV11/Hvβ21** | GGAGTAGACTCCACTCTCAAG |  |
|  | **TRBV2/Hvβ22** | GATCCGGTCCACAAAGCTGG |  |
|  | **TRBV13/Hvβ23** | ATTCTGAACTGAACATGAGCTCCT |  |
|  | **TRBV15/Hvβ 24** | GACATCCGCTCACCAGGCCTG |  |
| **T cell Receptor Beta Constant region (TRBC) / alias name** | **TRBC/Hcβ1α** | GGGTGTGGGAGATCTCTGC |  |
| **T cell Receptor Beta Junctional region (TRBJ) / alias names** | **TRBJ1.1/hJb1.1** | [6-FAM]TCTGGTGCCTTGTCCAAAGAAAGC |  |
|  | **TRBJ1.2/hJb1.2** | [6-FAM]CCTGTCCCCGAACCGAAGGTGTA |  |
|  | **TRBJ1.3/hJb1.3** | [6-FAM]CCAACTTCCCTCTCCAAAATATAT |  |
|  | **TRBJ1.4/hJb1.4** | [6-FAM]CTGGGTTCCACTGCCAAAAAACAG |  |
|  | **TRBJ1.5/hJb1.5** | [6-FAM]TCGAGTCCCATCACCAAAATGCTG |  |
|  | **TRBJ1.6/hJb1.6** | [6-FAM]CCTGGTCCCATTCCCAAAGTGGAG |  |
|  | **TRBJ2.1/hJb2.1** | [6-FAM]CCGTGTCCCTGGCCCGAAGAACTG |  |
|  | **TRBJ2.2/hJb2.2** | [6-FAM]CTAGAGCCTTCTCCAAAAAACAGC |  |
|  | **TRBJ2.3/hJb2.3** | [6-FAM]GGGTGCCTGGGCCAAAATACTGCG |  |
|  | **TRBJ2.4/hJb2.4** | [6-FAM]GGGTCCCGGCGCCGAAGTACTGAA |  |
|  | **TRBJ2.5/hJb2.5** | [6-FAM]CGCGTGCCTGGCCCGAAGTACTGG |  |
|  | **TRBJ2.6/hJb2.6** | [6-FAM]GCTGCCGGCCCCGAAAGTCAGGAC |  |
|  | **TRBJ2.7/hJb2.7** | [6-FAM]TGGTGCCCGGCCCGAAGTACTGCT |  |

Table S1**. Primers List.**

6’FAM are fluoriscinated primers; M stands for C or A, and Y stands for C or T)
