# Supplementary material for: Haemophilus parasuis (Glaesserella parasuis) as a Potential Driver of Molecular Mimicry and Inflammation in Rheumatoid Arthritis
Source: Front Med (Lausanne). 2021 Aug 17;8:671018. doi: 10.3389/fmed.2021.671018 (PMC8415917; doi:10.3389/fmed.2021.671018)
Supplement: Supplementary file 2 [file Table_2.docx]

| **Characteristics** | **RA** | **UPIA** | **Others** | **HC>30** | **HC<25** | **p^a^** | **p^b^** |
| --- | --- | --- | --- | --- | --- | --- | --- |
| Crevicular fluid | 27/47 (57.4) | 14/22 (63.6) | 21/31 (67.7) | 12/38 (31.6) | 87/131 (66.4) | P<0,01 | ns |
| Synovial tissue | 2/37 (5.4) | 0/16 | 0/25 | N/A | N/A |  |  |

Table S2. **Positive results for *Haemophilus parasuis* DNA**. All the values are considered as number of positives/total number (%). Others, other arthritides of different oriogins; RA, rheumatoid arthritis; UPIA, undifferentiated peripheral inflammatory arthritis. Statistical analyses are performed as described in methods significant differences are highlighted in bold displaying the p values: p^a^ = patients vs. HC>30, p^b^ = patients vs. HC<30.
