# Supplementary material for: Haemophilus parasuis (Glaesserella parasuis) as a Potential Driver of Molecular Mimicry and Inflammation in Rheumatoid Arthritis
Source: Front Med (Lausanne). 2021 Aug 17;8:671018. doi: 10.3389/fmed.2021.671018 (PMC8415917; doi:10.3389/fmed.2021.671018)
Supplement: Supplementary file 3 [file Table_3.docx]

| **Characteristics** | **RA** | **HC<25** |
| --- | --- | --- |
| **Number of sequences** | 90 | 74 |
| **Number of sequenced individuals** | 26 | 31 |
| **100% identity** | 1 | 0 |
| **>95% identity** | 10 | 13 |
| **>93% identity** | 14 | 19 |

Table S3. **Sequencing of 16S ribosomal RNA of *Haemophilus Parasuis***. The samples from 26 RA patients (previously positive at the PCR) and 31 young (age < 25 years) healthy controls have been sequenced for the Hps 16S ribosomal RNA. In about one out third of the patients and controls it has been possible to detect a sequence with an identity of more than 95% with the one reported in the literature ncbi (ref|NR_042879.1). In one RA patient it has been possible to find a sequence with an identity of 100%.
