# Supplementary material for: Haemophilus parasuis (Glaesserella parasuis) as a Potential Driver of Molecular Mimicry and Inflammation in Rheumatoid Arthritis
Source: Front Med (Lausanne). 2021 Aug 17;8:671018. doi: 10.3389/fmed.2021.671018 (PMC8415917; doi:10.3389/fmed.2021.671018)
Supplement: Supplementary file 4 [file Table_4.docx]

|  | Hps+ control | Parod. | #50 | | #40 | | #38 | | #76 | | #79 | | #77 | | #12 | | #18 | | #16 | | #14 | | #34 | | #13 | |
| --- | --- | --- | --- | --- | --- | --- | --- | --- | --- | --- | --- | --- | --- | --- | --- | --- | --- | --- | --- | --- | --- | --- | --- | --- | --- | --- |
| Access Number | MW 657623 | MW 657624 | | MW 657625 | | MW 657627 | | MW 657628 | | MW 657629 | | MW 657630 | | MW 657631 | | MW 657632 | | MW 657633 | | MW 657634 | | MW 657635 | | MW 657636 | | MW 657637 |
| IDENTITY | 100% | 100% | 100% | | 95% | | 95% | | 93% | | 95% | | 95% | | 95% | | 95% | | 95% | | 95% | | 95% | | 93% | |
|  | 730/730 | 730/730 | 720/720 | | 643/676 | | 674/708 | | 604/650 | | 720/759 | | 710/745 | | 690/726 | | 703/739 | | 711/736 | | 599/633 | | 677/703 | | 560/612 | |
|  |  |  |  | |  | |  | |  | |  | |  | |  | |  | |  | |  | |  | |  | |
| 461-3 | GTC | GTC | GTC | |  | |  | |  | |  | |  | |  | | AT- | | TC | |  | |  | |  | |
| 471-5 | AAGCA | AAGCA | AAGCA | |  | | AAGCA | |  | | AGTC | |  | |  | | AAGCA | | AAGCA | |  | | AAGCA | |  | |
| 482-3 | AA | AA | AA | |  | | AA | |  | |  | |  | |  | | AA | | AA | |  | | AA | |  | |
| 510 |  |  |  | |  | | G | |  | |  | | C | |  | | G | | G | |  | | G | |  | |
| 519-20 |  |  |  | |  | | GA | |  | |  | | AG | |  | | GA | | GA | |  | | GA | |  | |
| 535 | G | G | G | | A | | A | | A | | A | | A | |  | | A | | A | |  | | A | | G | |
| 543-4 | GT | GT | GT | | GT | | GT | | GT | | GT | | GT | |  | | GT | | GT | |  | | GT | | GT | |
| 560-1 | GG | GG | GG | | GG | | GG | | GG | | GG | | GG | |  | | G | | GG | |  | | GG | | GG | |
| 562-5 | TGAC | TGAC | TGAC | | CTAT | | CTAT | | CTAT | | ACTT | | CTAT | |  | | GACT | | CTAT | |  | | CTAT | | TGAA | |
| 575 | A | A | A | | G | | G | | G | | G | | G | |  | | G | | G | | G | | G | | G | |
| 583 | G | G | G | | T | | T | | T | | T | | T | | T | | T | | T | | T | | T | | T | |
| 589 | A | A | A | | G | | G | | G | | G | | G | | G | | G | | G | | G | | G | | G | |
| 591 | C | C | C | | C | | C | | C | | C | | C | | T | | C | | C | | C | | C | | C | |
| 597 | T | T | T | | C | | C | | C | | C | | C | | T | | C | | C | | C | | C | | T | |
| 599 | G | G | G | | G | | G | | G | | G | | G | | A | | G | | G | | G | | G | | G | |
| 604 | T | T | T | | T | | T | | T | | T | | T | | A | | T | | T | | T | | T | | TT | |
| 614 | T | T | T | | G | | G | | G | | G | | G | | G | | G | | G | | G | | G | | A | |
| 621 | T | T | T | | T | | T | | T | | T | | T | | G | | A | | T | | T | | T | | T | |
| 622 | T | T | T | | A | | A | | A | | A | | A | | T | | G | | A | | A | | A | | A | |
| 621-4 | TTGC | TTGC | TTGC | | TAGC | | TAGC | | TAGC | | TAGC | | TAGC | | TTGC | | AGTC | | TAGC | | TAGC | | TAGC | | TACT | |
| 632 | T | T | T | | C | | C | | C | | C | | C | | C | | C | | C | | C | | C | | C | |
| 687 | G | G | G | | G | | G | | G | | G | | G | | G | | G | | G | | G | | G | | A | |
| 703 | G | G | G | | G | | G | | G | | G | | G | | G | | G | | G | | G | | G | | G | |
| 719-20 | AA | AA | AA | | TG | | TG | | TG | | TG | | TG | | TG | | TG | | TG | | TG | | TG | | TG | |
| 738 | G | G | G | | G | | G | | G | | G | | G | | G | | G | | G | | G | | G | | T | |
| 753 | A | A | A | | A | | A | | A | | A | | A | | A | | A | | A | | A | | A | | A | |
| 758-60 | GGA | GGA | GGA | | GGA | | GGA | | GGA | | GGA | | GGA | | GGA | | GGA | | GGA | | GGA | | GGA | | GGA | |
| 764 | G | G | G | | G | | G | | G | | G | | G | | G | | G | | G | | G | | G | | G | |
| 795 | T | T | T | | T | | T | | T | | T | | T | | T | | T | | T | | T | | T | | T | |
| 797 | G | G | G | | G | | G | | G | | G | | G | | G | | G | | G | | G | | G | | A | |
| 813-6 | TTAG | TTAG | TTAG | | TTTA | | TTTA | | CTTG | | CTTG | | TTTG | | TT-G | | TTA- | | TTTA | | TTTA | | TTTA | | TTAA | |
| 818-9 | GC | GC | GC | | GC | | GC | | GC | | GC | | GC | | GC | | GC | | GC | | GC | | GC | | GC | |
| 829-36 | GTAGCTAA | GTAGCTAA | GTAGCTAA | | GTAGCTAA | | GTAGCTAA | | GTAGCTAA | | GAAGCTAA | | GTAGCTAA | | GTAGCTAA | | GTAGCTAA | | GTAGCTAA | | GTAGCTAA | | GTAGCTAA | | GAAGCTAA | |
| 852 | G | G | G | | G | | G | | G | | G | | G | | G | | G | | G | | N | | G | | C | |
| 908 | A | A | A | | A | | A | | A | | C | | A | | A | | A | | A | | A | | A | | A | |
| 919 | C | C | C | | C | | C | | C | | CC | | C | | C | | C | | C | | C | | C | | C | |
| 943 | C | C | C | | C | | C | | C | | CC | | C | | C | | C | | C | | C | | C | | C | |
| 971-76 | TAAGAA | TAAGAA | TAAGAA | | ATGGAA | | ATGGAA | | ATGGGAA | | ATGGAA | | ATGGAA | | ATGGAA | | ATGGAA | | ATGGAA | | CATGGAA | | ATGGAA | | GTGGAA | |
| 977-982 | GAACTC | GAACTC | GAACTC | | TCTTGT | | TCTTGT | | TCTTGT | | TCTTGT | | TCNTGT | | TCTTGT | | TCTTGT | | TCTTGT | | TCTTGT | | TTCTGT | | TCCTGT | |
| 989-994 | GAGTTT | GAGTTT | GAGTTT | | ACGGGA | | ACGGGA | | ATGAGA | | ATGAGA | | ATGAGA | | ATGAGA | | ATGAGA | | ACGGGA | | ACGGGA | | ACGGAA | | ACGGGA | |
| 1009-1111 | TTA | TTA | TTA | | CAT | | CAT | | CAT | | CAT | | CAT | | CAT | | CAT | | CAT | | CATGGAA | | CAT | | CAT | |
| 1008 | C | C | C | | C | | T | | C | | A | | C | | A | | C | | C | | C | | C | | C | |
| 1034 | C | C | C | | C | | C | | C | | C | | C | | C | | C | | C | | C | | C | | T | |
| 1043 | G | G | G | | G | | G | | G | | G | | G | | G | | G | | G | | G | | G | | A | |
| 1079 | G | G | G | | G | | G | | G | | G | | G | | G | | G | | G | | G | | G | | C | |
| 1094 | T | T | T | | T | | T | | T | | T | | T | | T | | T | | T | | T | | T | | G | |
| 1130 | C | C | C | | C | | C | | CC | | C | | C | | C | | C | | C | | C | | C | | C | |
| 1134 | A | A | A | | G | | G | | G | | G | | G | | G | | G | | G | | G | | G | | A | |
| 1144 | T | T | T | | C | | C | | C | | C | | C | | C | | C | | C | | C | | C | | C | |

Table S4

**Comparison among sequences of 16S ribosomal RNA of *Haemophilus Parasuis* and 13 different RA patients.** The samples from 12 RA patients (previously positive at the PCR) and with a sequence of identity >91% are displayed in comparison with the ref. Seq. of the *Hps* 16S ribosomal RNA gene from ncbi. All the different segments were compared to first column that contains the positive control of *Hps* 16S gene. (HPS+ control= MW657623; Parod.= MW657624; #50= MW657625; #40= MW657627; #38= MW657628; #76= MW657629; #79= MW657630; #77= MW657631; #12= MW657632; #18= MW657633; #16= MW657634; #14= MW657635; #34= MW657636; #13= MW657637; submitted to GenBank SUB9141976).
