# Supplementary material for: Haemophilus parasuis (Glaesserella parasuis) as a Potential Driver of Molecular Mimicry and Inflammation in Rheumatoid Arthritis
Source: Front Med (Lausanne). 2021 Aug 17;8:671018. doi: 10.3389/fmed.2021.671018 (PMC8415917; doi:10.3389/fmed.2021.671018)
Supplement: Supplementary file 5 [file Table_5.docx]

| **Characteristics** | **ERA patients** |
| --- | --- |
| **N.** | 13 |
| **Age**, (years) | 58.8 ± 17.4 |
| **Sex**, n° female, (%) | 9.0 (69.2) |
| **Ab** positive (%) | 8 (61.5) |
| **ESR** (mm/1^^^hour) | 68.5 ± 34.9 |
| **CRP** (mg/l) | 50.1 ± 56.3 |
| **TJC** | 13.6 ± 10.7 |
| **SJC** | 10.5 ± 8.7 |
| **DAS** | 3.9 ± 1.2 |
| **HAQ** | 1.4 ± 0.9 |
| **Symptoms’ Duration** (months) | 5.27 ± 1.09 |
| **HLA-DRB1*01** n. (%) | 2 (15.4) |
| **HLA-DRB1*04** n. (%) | 7 (53.8) |
| **HLA-DRB1*11** n. (%) | 4 (30.8) |

Table S5. **Demographic, immunological and clinical characteristics of ERA patients at diagnosis**. Values are mean ± standard deviation unless otherwise indicated. ERA: early rheumatoid arthritis; ACPA: anti-citrullinated peptide antibodies; RF: rheumatoid factor; ESR: erythrocyte sedimentation rate; CRP: C-reactive protein; TJC: tender joint count; SJC: swollen joint count; DAS: disease activity score; HAQ: Health Assessment Questionnaire. HLA: histocompatibility Leucocyte Antigen.
