# Supplementary material for: Haemophilus parasuis (Glaesserella parasuis) as a Potential Driver of Molecular Mimicry and Inflammation in Rheumatoid Arthritis
Source: Front Med (Lausanne). 2021 Aug 17;8:671018. doi: 10.3389/fmed.2021.671018 (PMC8415917; doi:10.3389/fmed.2021.671018)
Supplement: Supplementary file 6 [file Presentation_1.pdf]

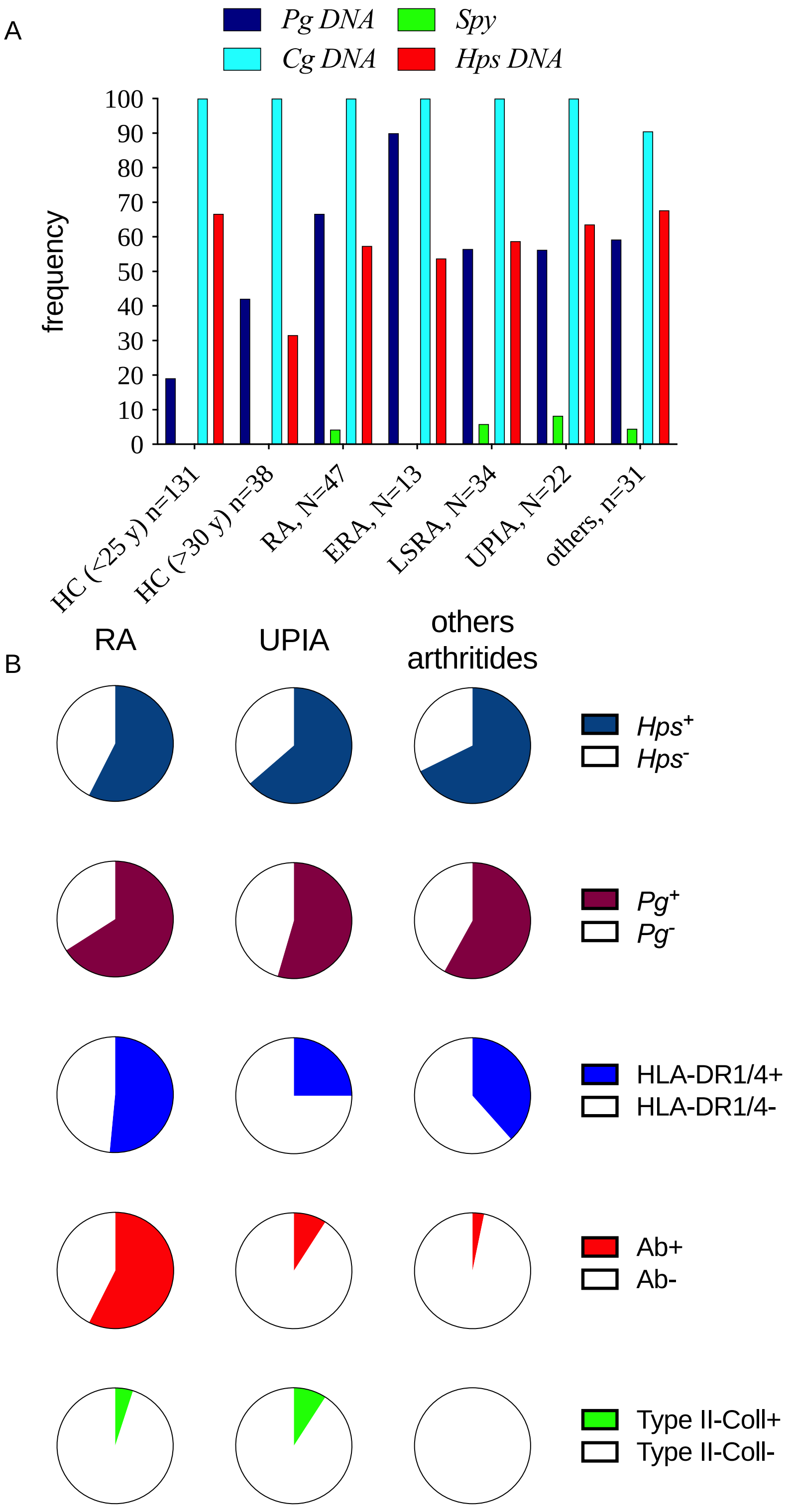

Supplementary Figure 1.
Hps, Pg, Spy and *Cg* detection: comparison and correlation.
This figure completes figure 1. A) Each bar represents the % of the DNA samples obtained from the crevicular fluid of the patients and healthy controls positive for the PCR test for 16S ribosomal RNA of *Haemophilus parasuis* [27-29] (red bars) *Streptococcus pyogenes* (green bars), *Capnocytophaga* spp (light blue bars) and *Porphyromonas gingivalis*[30] (violet bars). *Streptococcus pyogenes* was detected as described by authors[31]. *Capnocytophaga spp* detection was performed as detailed in[32]. B) Pie charts of patients affected by RA, UPIA or other arthritides; eahc row represents a different positivity for *Hps*, *Pg*, HLA-DRB1*01 and/or *04, ACPA and/or RF and anti- type 2 Collagen. (αCollagen =anti-Collagen type 2 antibodies; ACPA=Anti-citrullinated protein antibodies; RF= Rheumatoid factor; DR1 and DR4= HLA-DRB1*01 and 04 respectively.

#50

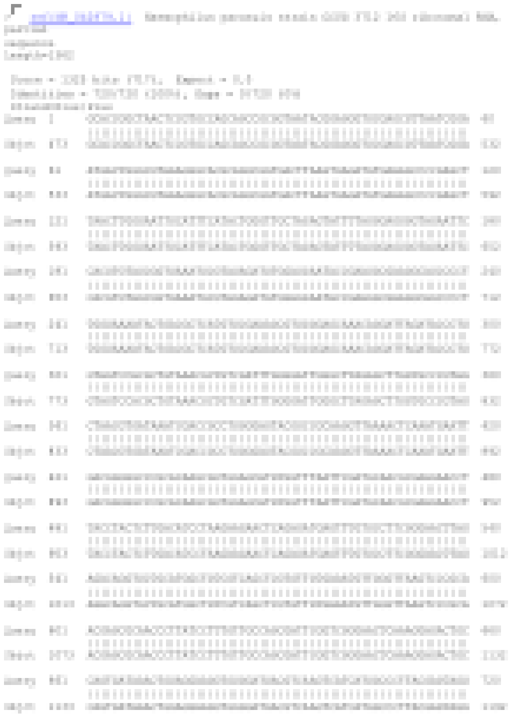

#38

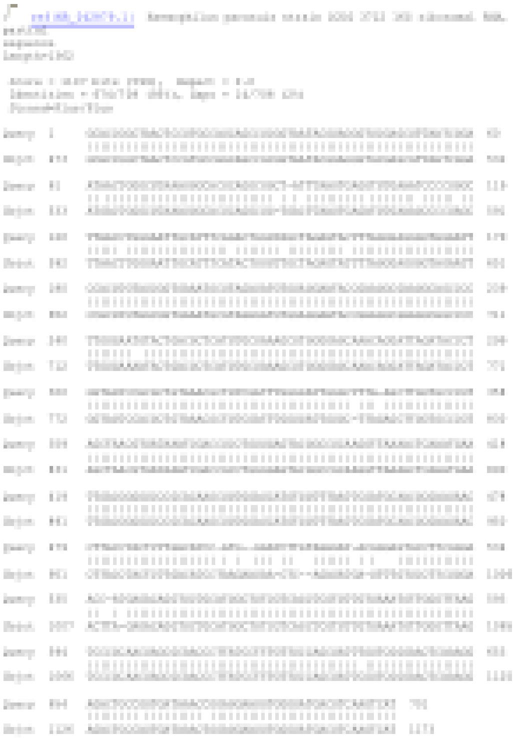

#40

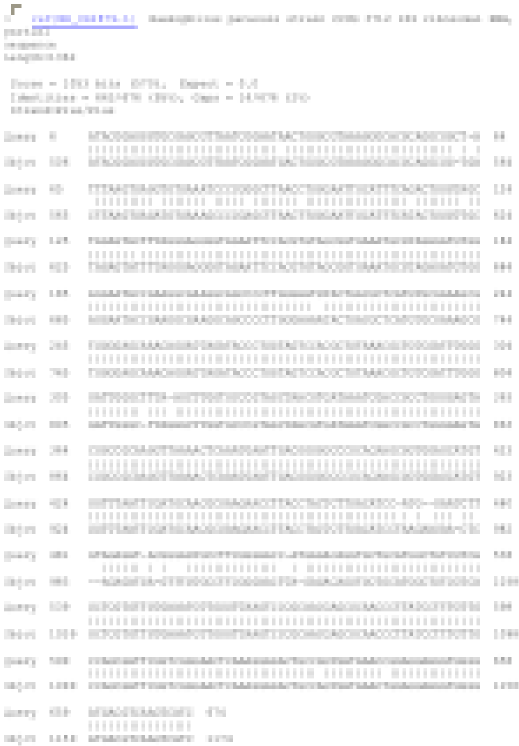

#76

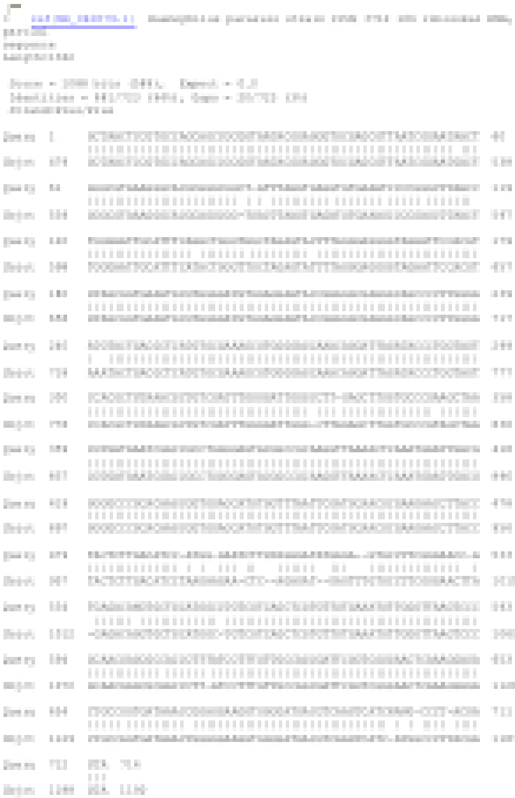

Supplementary Figure 2.

Examples of gene Homology. The figure displays the gene homologies between 4 exemplificative sequenced samples from RA patients and 16S ribosomal RNA gene of Hps. Sample of patient number #50 has a 100% identity.

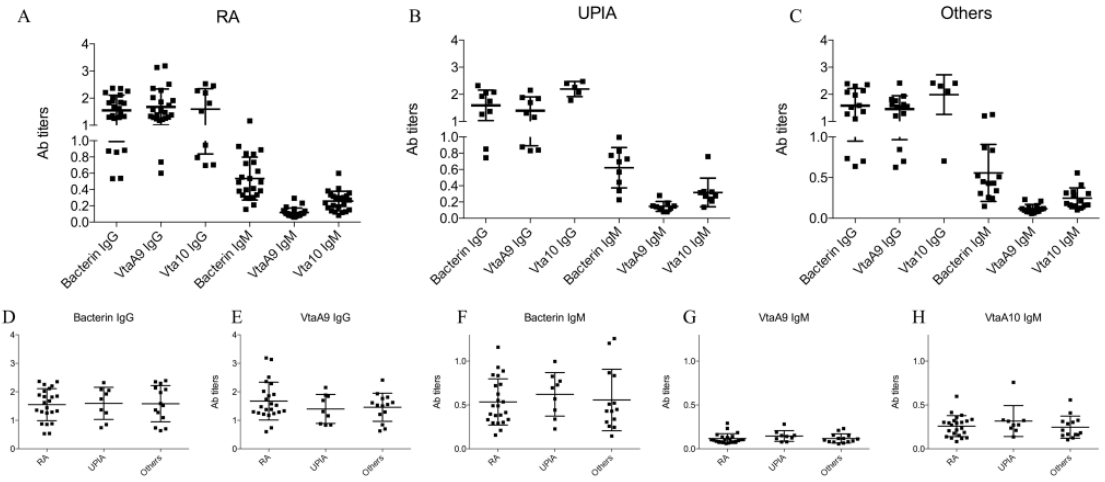

Supplementary Figure 3.

IgM and IgG titers specific for Hps in RA, UPIA and other patients affected by arthritides of different origin. Each symbol represents a patient. Patients are displayed in three graphs on the basis of the diagnosis (A-C) or in five graphs based of Bacterin, VtaA9 and VtaA10 Ab titers (D-H).

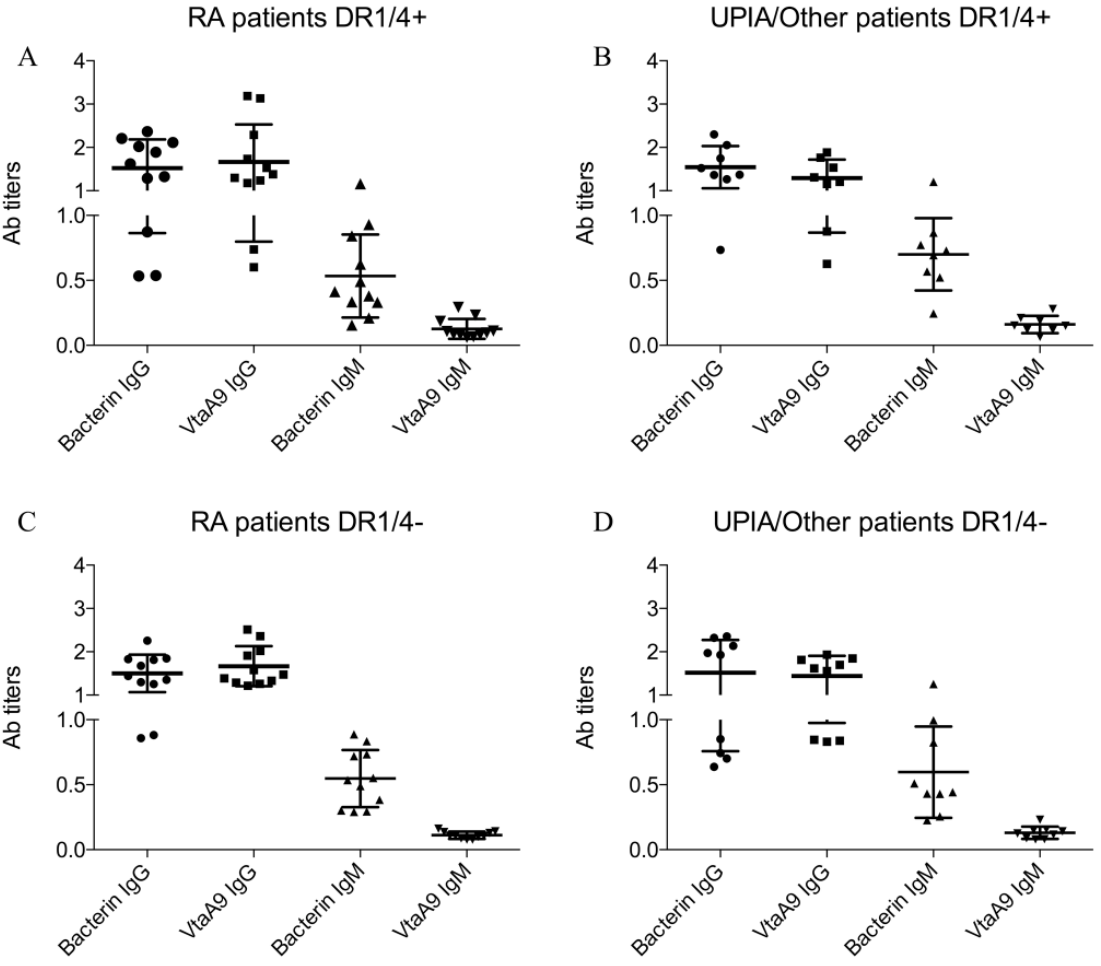

Supplementary Figure 4.

IgM and IgG titers specific and HLA-DR4/DR1.
Each symbol represents a patient. Patients are displayed in 4graphs on the basis of the diagnosis RA patients (A and C) or UPIA/Others patients (B and D) and on the basis for the positivity of at least a HLA-DR4 or HLA-DR1 allele.

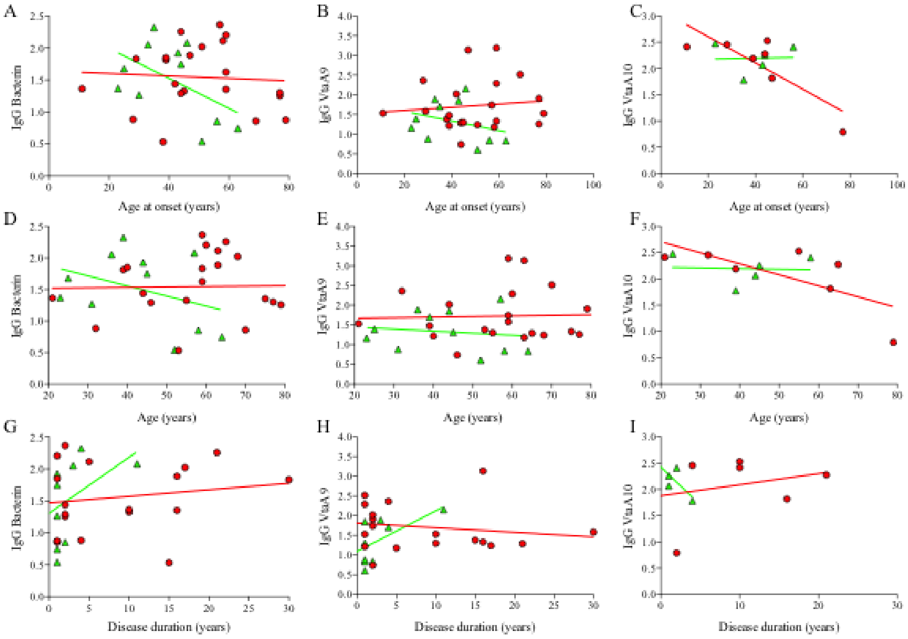

Supplementary Figure 5.

IgG titers in relation to the Age at the onset, the age and the disease duration of RA and UPIA patients. Each symbol represents a patient (red circles for RA and green triangle for UPIA). There are significant direct correlations that are detectable in C for RA patients (r=-0.92 and p=0.004) and F for RA patients (r=-0.76 and p=0.05). The other correlations have not significant values.

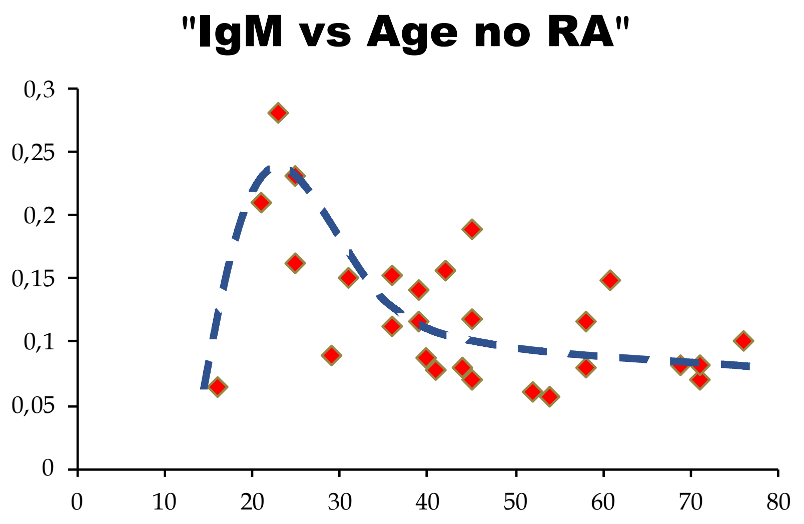

Supplementary Figure 6.

IgM titers specific for Hps in patients affected by UPIA or arthritides of different origin. IgM titers are displayed in function of age. RA patients are excluded by this analysis. Highest levels of IgM specific for VtaA10 were found in subjects in their 3rd decade of life. The only fifteen years-old patients showed the lowest levels of OD for both IgM and IgG specific for VtaA10.

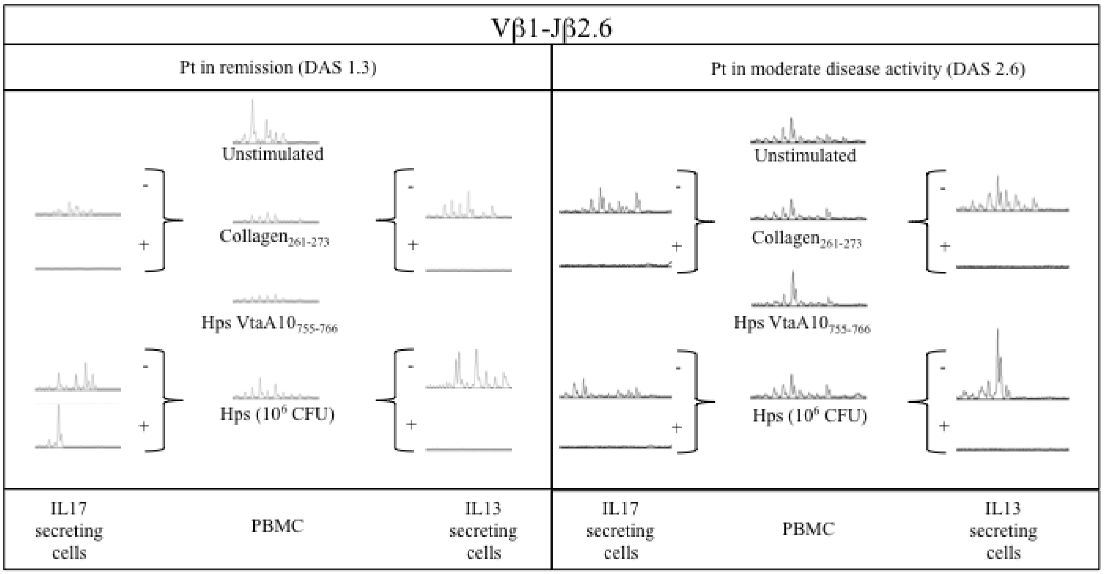

Supplementary Figure 7.

Vβ1-Jβ2.6 carrying unsorted and sorted T cells of two DR4+ ERA patients.
PBMC samples and sorted IL17 and IL13 secreting T cells from 2 ERA patients with different disease activity (one in remission on the left and the other in active phase of disease) where compared through Immunoscope analysis. The presence of an antigen-specific expansion is displayed with a perturbation of the gaussian or the presence of a single peak where possible the comparison could be done through the rate between the area of the same length peaks of two different samples (RSI or rate stimulation index).

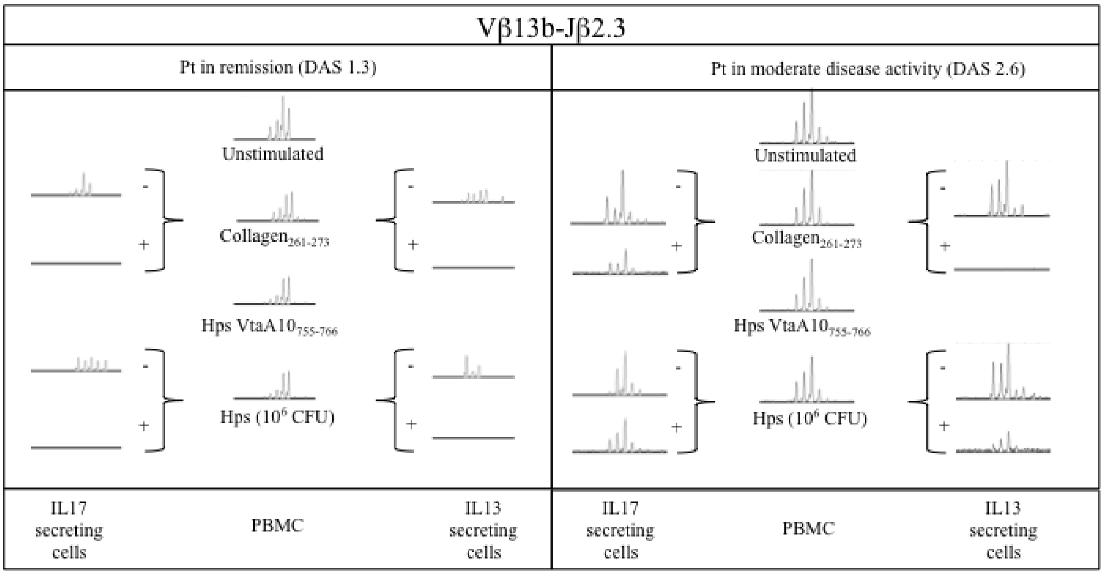

Supplementary Figure 8.

Vβ13b-Jβ2.3 carrying unsorted and sorted T cells of two DR4+ ERA patients.
PBMC samples and sorted IL17 and IL13 secreting T cells from 2 ERA patients with different disease activity (one in remission on the left and the other in active phase of disease) where compared through Immunoscope analysis. The presence of an antigen-specific expansion is displayed with a perturbation of the gaussian or the presence of a single peak where possible the comparison could be done through the rate between the area of the same length peaks of two different samples (RSI or rate stimulation index).

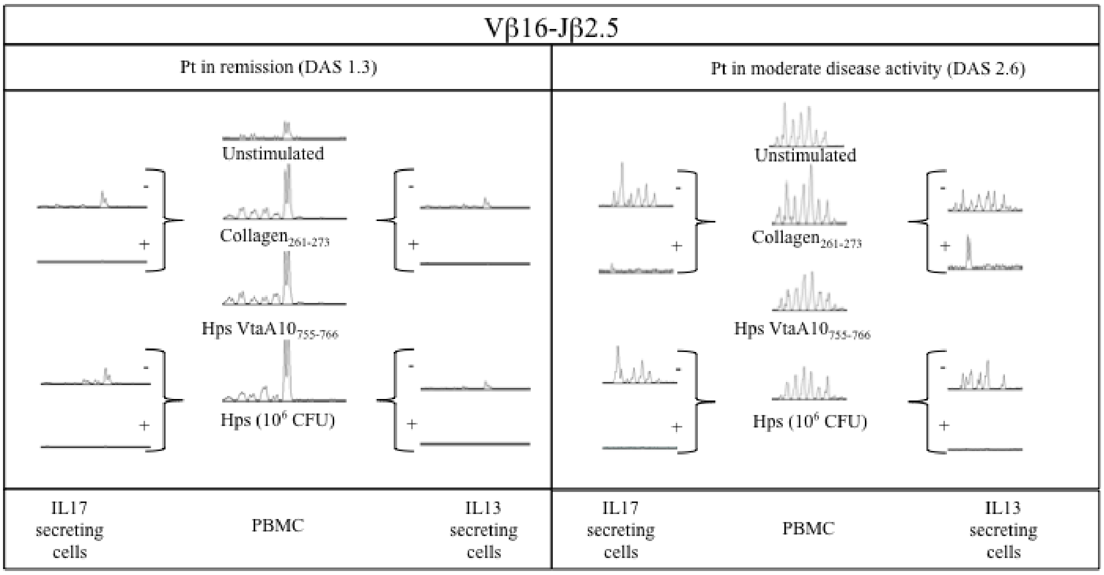

Supplementary Figure 9.

Vβ16-Jβ2.5 carrying unsorted and sorted T cells of two DR4+ ERA patients.
PBMC samples and sorted IL17 and IL13 secreting T cells from 2 ERA patients with different disease activity (one in remission on the left and the other in active phase of disease) where compared through Immunoscope analysis. The presence of an antigen-specific expansion is displayed with a perturbation of the gaussian or the presence of a single peak where possible the comparison could be done through the rate between the area of the same length peaks of two different samples (RSI or rate stimulation index).

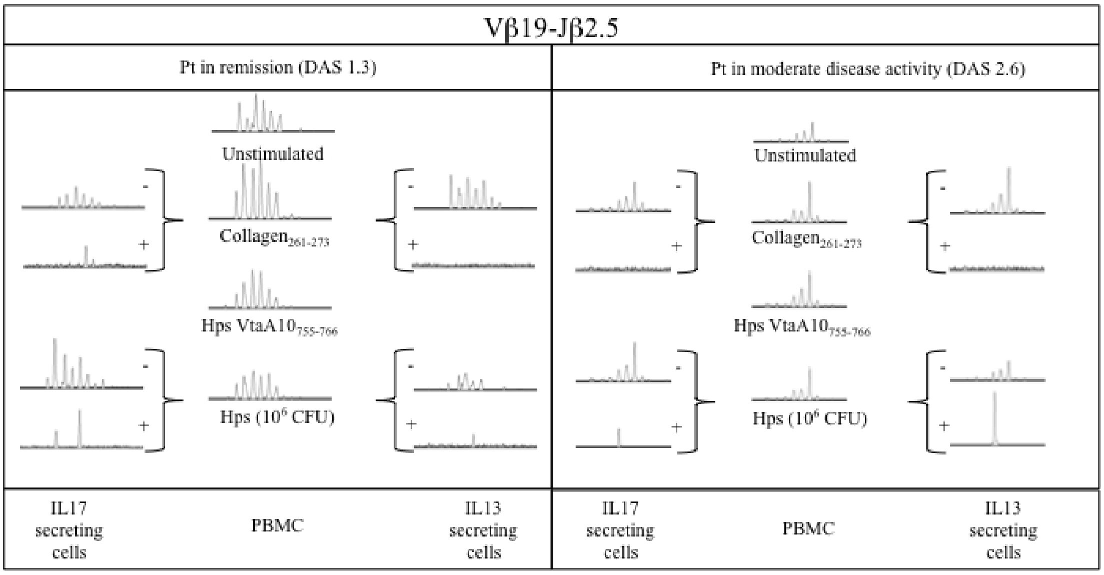

Supplementary Figure 10.

Vβ19-Jβ2.5 carrying unsorted and sorted T cells of two DR4+ ERA patients.

PBMC samples and sorted IL17 and IL13 secreting T cells from 2 ERA patients with different disease activity (one in remission on the left and the other in active phase of disease) where compared through Immunoscope analysis. The presence of an antigen-specific expansion is displayed with a perturbation of the gaussian or the presence of a single peak where possible the comparison could be done through the rate between the area of the same length peaks of two different samples (RSI or rate stimulation index).
